# High-resolution melting analysis identifies reservoir hosts of zoonotic *Leishmania* parasites in Tunisia

**DOI:** 10.1186/s13071-021-05138-x

**Published:** 2022-01-08

**Authors:** Moufida Derghal, Abir Tebai, Ghofrane Balti, Hajer Souguir-Omrani, Jomaa Chemkhi, Adel Rhim, Ali Bouattour, Ikram Guizani, Youmna M’Ghirbi, Souheila Guerbouj

**Affiliations:** 1 Laboratoire d’Epidémiologie Moléculaire Et Pathologie Expérimentale Appliquée Aux Maladies Infectieuses (LR16IPT04), Institut Pasteur de Tunis, Université Tunis El Manar, Tunis, Tunisia; 2 Laboratoire d’épidémiologie Et Microbiologie Vétérinaire (LR16IPT03), Institut Pasteur de Tunis, Université Tunis El Manar, Tunis, Tunisia; 3 Laboratoire Des Virus, Vecteurs Et Hôtes (LR20IPT02), Institut Pasteur de Tunis, Université Tunis El Manar, Tunis, Tunisia; 4 Faculté Des Sciences de Tunis, Université Tunis El Manar, Tunis, Tunisia

**Keywords:** *Leishmania*, High-resolution melting analysis, 7SL RNA, HSP70, Reservoir host, Tunisia

## Abstract

**Background:**

Leishmaniasis is endemic in Tunisia and presents with different clinical forms, caused by the species *Leishmania infantum*, *Leishmania major*, and *Leishmania tropica*. The life cycle of *Leishmania* is complex and involves several phlebotomine sand fly vectors and mammalian reservoir hosts. The aim of this work is the development and evaluation of a high-resolution melting PCR (PCR-HRM) tool to detect and identify *Leishmania* parasites in wild and domestic hosts, constituting confirmed (dogs and *Meriones* rodents) or potential (hedgehogs) reservoirs in Tunisia.

**Methods:**

Using in vitro-cultured *Leishmania* isolates, PCR-HRM reactions were developed targeting the 7SL RNA and HSP70 genes. Animals were captured or sampled in El Kef Governorate, North West Tunisia. DNA was extracted from the liver, spleen, kidney, and heart from hedgehogs (*Atelerix algirus*) (*n* = 3) and rodents (*Meriones shawi*) (*n* = 7) and from whole blood of dogs (*n* = 12) that did not present any symptoms of canine leishmaniasis. In total, 52 DNA samples were processed by PCR-HRM using both pairs of primers.

**Results:**

The results showed melting curves enabling discrimination of the three *Leishmania* species present in Tunisia, and were further confirmed by Sanger sequencing. Application of PCR-HRM assays on reservoir host samples showed that overall among the examined samples, 45 were positive, while seven were negative, with no *Leishmania* infection. *Meriones shawi* were found infected with *L. major*, while dogs were infected with *L. infantum*. However, co-infections with *L. major*/*L. infantum* species were detected in four *Meriones* specimens and in all tested hedgehogs. In addition, multiple infections with the three *Leishmania* species were found in one hedgehog specimen. Sequence analyses of PCR-HRM products corroborated the *Leishmania* species found in analyzed samples.

**Conclusions:**

The results of PCR-HRM assays applied to field specimens further support the possibility of hedgehogs as reservoir hosts of *Leishmania*. In addition, we showed their usefulness in the diagnosis of canine leishmaniasis, specifically in asymptomatic dogs, which will ensure a better evaluation of infection extent, thus improving elaboration of control programs. This PCR-HRM method is a robust and reliable tool for molecular detection and identification of *Leishmania* and can be easily implemented in epidemiological surveys in endemic regions.

**Graphical Abstract:**

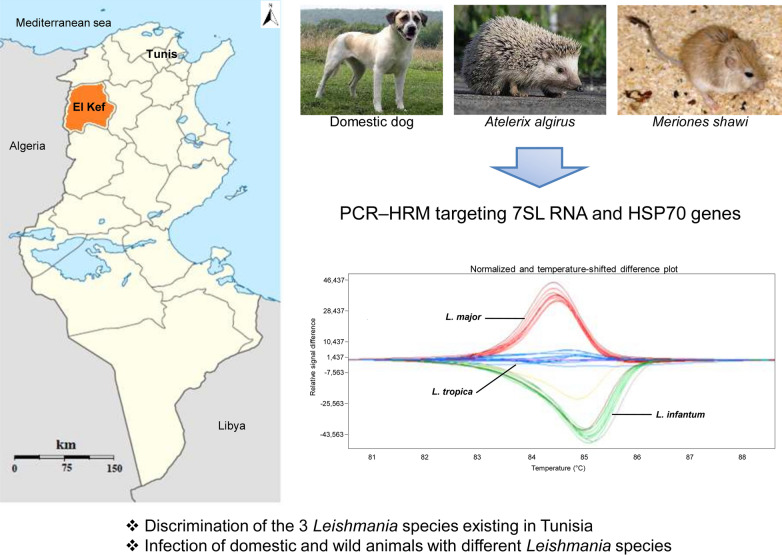

**Supplementary Information:**

The online version contains supplementary material available at 10.1186/s13071-021-05138-x.

## Background

Leishmaniasis represents a group of protozoan parasitic diseases caused by species belonging to the genus *Leishmania.* It is endemic and occurs in more than 98 countries on five continents, with the exception of New Zealand and the southern Pacific [1]. So far, around 31 out of 53 identified *Leishmania* species have been reported to infect mammals, 20 of which cause various clinical manifestations in humans [1]. Worldwide, at least 70 species of wild and domestic animals, including dogs, opossums, bats, anteaters, sloths, marsupials, rodents, and hyraxes, have been identified as confirmed or potential reservoirs of *Leishmania* parasites [2]. In Tunisia, four clinical forms exist and are caused by different parasite species and hosted by various reservoirs. Visceral leishmaniasis (VL) is caused by *Leishmania infantum*, and its main reservoir is the dog [3], whereas for the sporadic cutaneous form (SCL) also due to this species, dogs are considered to be reservoirs [4]. Two other cutaneous forms exist in Tunisia, the highly endemic zoonotic cutaneous leishmaniasis (ZCL), which is caused by *Leishmania major* [4], and the chronic cutaneous leishmaniasis (CCL) caused by *Leishmania tropica* (syn. *Leishmania killicki*) [5]. These forms have as respective reservoirs rodents (*Psammomys* and *Meriones*) and gundies (*Ctenodactylus gundi*) [4, 6]. Recently, hedgehogs were identified as potential reservoirs of *L. major* in Iran [7]. A few years ago, in 2014, these animals were reported to be infected by *L. major* in both *Atelerix algirus* and *Paraechinus aethiopicus* in Algeria [8]. While in 2015, *A. algirus* hedgehogs were found to be co-infected by the two species *L. major*, and *L. infantum* in EL Kef Governorate, a region located in North West Tunisia and known to be endemic for SCL due to *L. infantum* [9]. These findings were later confirmed, suggesting the potential role of this animal as a reservoir host of leishmaniasis in Tunisia [10].

The methods used in these studies included various classical PCR techniques that target either non-coding or coding regions of the *Leishmania* genome [11]. However, these assays have multiple disadvantages, particularly regarding time consumption in post-PCR processing, including electrophoresis on agarose gels, confirmation with additional treatment by restriction fragment length polymorphism (RFLP), and sequencing, thus expending resources to overcome the challenge of spurious product generation during gene amplification with low sensitivity and high cost. Therefore, the development of other tools with higher sensitivity constitutes a better option for accurate detection and identification of *Leishmania* species in field-caught animals. Indeed, high-resolution melting PCR (PCR-HRM), which is a very sensitive amplification technique, enables the direct characterization of amplicons in a closed tube assay and does not require additional precautions to prevent crossover of PCR products [12]. It measures changes in the fluorescence intensity of a DNA-intercalating dye during dissociation from double-stranded DNA to single-stranded DNA, thus detecting single nucleotide polymorphism (SNP) [13]. In the last few years, this technique has become widely used to ensure better specificity, sensitivity, and reproducibility in molecular diagnostics of multiple diseases [12, 13]. To achieve robust *Leishmania* parasite detection and increase the sensitivity of the assays, previous studies applying PCR-HRM used markers present in abundant copy numbers in the *Leishmania* genome, including the heat shock protein 70 gene (HSP70) [14, 15], ITS1 sequences [14, 16], 7SL RNA gene [17], and LACK gene [18].

The aim of this study is the development and evaluation of molecular tools based on PCR-HRM to study *Leishmania* infection in wild and domestic mammal hosts constituting confirmed (dogs and rodents) and potential (hedgehogs) reservoirs of leishmaniasis in an endemic focus in North West Tunisia.

## Methods

### Study site, animal identification, and sample collection

The study was carried out in the governorate of EL Kef, which is situated in North West Tunisia and is known as an active focus of SCL due to *L. infantum* species. From 2015 to 2019 during leishmaniasis transmission seasons, field trips to three localities within this region (KaletSnen, Dahmani, and Zaafrann in Oued Souani) allowed us to capture different specimens of rodents (*Meriones shawi*; *n* = 7) and hedgehogs (*n* = 3). Additionally, blood samples and ocular/gum swabs were collected from dogs (*n* = 43) from the same region. After their transfer to the laboratory, hedgehogs and rodents were humanely euthanized and the corresponding genus and species were determined based on external morphological criteria [19, 20]. Measurements of the body, head, ear, hind foot, and tail were systematically recorded for each specimen (Additional file 1: Table S1). Different types of samples were taken from each specimen, including liver, spleen, kidney, heart, lymph node, and skin. However, according to possibilities of sampling, gum swab, eye swab, blood, urine, fat, peritoneum, bone marrow, and liver nodules were also collected from some specimens (Additional file 2: Table S2). All samples were conserved in liquid nitrogen until later analysis. In addition, 27 *Leishmania* strains isolated from in vitro-cultured parasites representing the Old World species *L. major*, *L. infantum*, and *L. tropica* (Additional file 3: Table S3) were used to test the specificity of the developed PCR-HRM assays.

### DNA extraction

Whole genomic DNA was extracted from the biological material collected from dogs (blood [n = 43] and ocular/gum swabs [n = 52]) as well as organ samples and biological liquids of *Meriones* (n = 45) and hedgehogs (n = 31) (Additional file 2: Table S2). All extractions were realized by the conventional phenol/chloroform method [21] and all DNA was suspended in 10 mM Tris-1 mM EDTA p H8 solution and conserved at +4 °C. Extracted DNA was quantified using a Qubit Fluorometer (Invitrogen, France). Dilutions in DNAase/RNAase-free water at 50 or 20 ng/µl were prepared and used in PCR reactions.

### Development of PCR-HRM

#### Choice of target sequences and primer design

Two gene sequences were targeted in this study. First, primers targeting a conserved region coding for the 7SL RNA subunit were used, as described previously [17]. This allowed amplification of a 119-base-pair (bp) DNA fragment. The second pair of primers targeting the HSP70 gene was designed here. Thus, using Clustal X, HSP gene sequences from *L. major*, *L. infantum*, and *L. tropica* were aligned and primers were designed in conserved regions flanking a 280-bp polymorphic internal region containing multiple SNPs, using the Primer3Plus software program (http://www.bioinformatics.nl/cgibin/primer3plus/primer3plus.cgi). Primer-BLAST (http://www.ncbi.nlm.nih.gov/tools/primer-blast/) was also used to check the primers’ sequence specificity.

#### Conventional PCR assays

Prior to *Leishmania* DNA detection with PCR-HRM, both pairs of primers, 7SL RNA (CJ7SL/QRT7SL) and HSP70 (F_hsp70/R_hsp70), were tested by conventional PCR on DNA extracted from in vitro-cultured parasites representing Old World species *L. major* (MHOM/TN/2011/EMPA10), *L. infantum* (MHOM/TN/94/LV50), and *L. tropica* (MHOM/IQ/65/L75) (Additional file 3: Table S3). PCR amplifications were set up using different concentrations of MgCl_2_, a gradient of annealing temperatures, and addition of dimethyl sulfoxide (DMSO) at 5% and 10%. In addition, their ability to detect *Leishmania* infection was tested using a collection of animal samples by conventional PCR primer pairs with the reaction conditions set up earlier. Absence of amplification found in several cases was monitored by repeating the PCR up to three times or, occasionally, by a re-amplification step of the first product. In order to assess the quality of newly extracted DNA, a PO (acidic ribosomal phosphoprotein) PCR targeting a 470-bp fragment in a conserved region of a mammalian gene [22] was carried out for all samples. To monitor for possible contamination, a negative control (no DNA) was introduced in all PCR reactions. PCR conditions, cycling, and primer sequences used are reported in Additional file 4: Table S4.

#### DNA sequencing and analysis

Fragments generated by conventional PCR targeting 7SL RNA and HSP70 genes from each *Leishmania* strain used (*L. major*, *L. infantum*, *L. tropica*) as well as from animal samples were purified with phosphatase and exonuclease enzymes (Thermo Fisher Scientific, USA) and sequenced in both directions using the BigDye Terminator v3.1 Cycle Sequencing Kit (Applied Biosystems, USA). Sequencing was performed on an ABI 3500 sequencer (Applied Biosystems, USA). The generated sequences were visualized and manually corrected using BioEdit software, and then submitted to BLAST (basic local alignment search tool) analysis for homology searches using the National Center for Biotechnology Information (NCBI) server (http://blast.ncbi.nlm.nih.gov/). Direct sequencing assays were also carried out on PCR-HRM amplified products from animal samples, in order to confirm their *Leishmania* species identity and eventually validate ambiguous cases. The same sequencing procedure and analysis was followed as mentioned above.

#### *PCR*-*HRM development*

PCR-HRM development was first performed on control DNA extracted from in vitro-cultured strains of *Leishmania* species (*L. major*, *L. infantum*, and *L. tropica*). This was carried out with 7SL RNA and HSP70 gene primers by varying several parameters including a program with or without touchdown regarding the primer annealing temperatures, addition or no addition of DMSO at 5% or 10%, and DNA concentrations. Then, in order to ensure clear discrimination between *Leishmania* species and to determine the accuracy and reliability of the method herein reported, developed conditions were applied on a panel of 27 *Leishmania* strains corresponding to several strains of *L. major*, *L. infantum*, and *L. tropica* species (Additional file 3: Table S3). The sensitivity of the PCR-HRM targeting 7SL RNA and HSP70 genes was determined using tenfold dilutions of DNA from each reference *Leishmania* isolate, representing *L. infantum* (MHOM/TN/94/LV50), *L. major* (MHOM/TN/2011/EMPA10), and *L. tropica* (MHOM/IQ/65/L75). DNA amounts used were 20 ng and a range of 10 ng to 0.0001 ng.

PCR-HRM reactions were carried out in a final volume of 20 µl containing 20 ng of DNA, 2× LightCycler^®^ 480 High Resolution Melting Master Mix (Roche, France), 25 mM MgCl_2_ and 0.4 µM of each primer. A LightCycler^®^ 480 protocol (Roche, France) was then used, which included the following program: pre-incubation consisting of a denaturation step at 95 °C for 10 min followed by 45 cycles of amplification. The cycles comprised of the following steps: 95 °C for 10 s, then a touchdown protocol for 15 s covering a range of annealing temperatures from 60 and 53 °C by decreasing 0.2 °C at every cycle, followed by elongation at 72 °C for 10 s. To obtain the melting curves, the PCR program was followed by a melting program, in which a dissociation of the amplicon was performed at 95 °C for 1 min, then rapid cooling at 40 °C for 1 min, followed immediately by a fusion step at 60 °C for 1 s, with ramp rates at 4.4, 2.2, and 1 °C/s, respectively for each step. Finally, melting curves were obtained by re-association at 95 °C, with a ramp rate of 0.02 °C/s and 25 acquisitions/°C, which are sufficient to result in a resolution appropriate for HRM analysis, as recommended by the manufacturer.

Results were analyzed using the LightCycler^®^ 480 SW 5.1 software package, including the gene scanning module (version 1.5.0), provided by the manufacturer. For each experiment, PCR efficiency was evaluated using the threshold cycle (Ct) and melting curves were normalized according to obtained reaction values. Analysis of HRM results was realized by comparison of the obtained melting profiles to those corresponding to reference species (*L. major*, *L. infantum*, and *L. tropica*), which were systematically added as positive controls, in every reaction.

### Validation of the PCR-HRM tool on wild and domestic mammals

The developed PCR-HRM was validated on DNA extracted from different samples (n = 52) from the studied animals: *Meriones* (*n* = 7), hedgehogs (*n* = 3), and dogs (*n* = 12) (Additional file 2: Table S2). Reaction conditions and programming, and melting curves normalization and analysis were performed as described earlier.

## Results

### Development of PCR-HRM

#### Conventional PCR assays

The specificity of 7SL (CJ7SL/QRT7SL) and HSP70 (F_hsp70/R_hsp70) primers was determined by performing conventional PCR amplification, using genomic DNA extracted from in vitro-cultured parasites of three *Leishmania* species (*L. infantum*, *L. major*, and *L. tropica*). Amplified fragments at the expected sizes (119 bp and 280 bp, respectively) were found (Additional file 5: Fig. S1). In order to get a preliminary idea about *Leishmania* infection in collected animals (*Meriones*, hedgehogs, and dogs), these conventional PCRs were applied on the different samples, in addition to inclusion of *L. infantum*, *L. major*, and *L. tropica* representative species as positive controls. It is worth noting here that prior to the application of conventional 7SL and HSP70 PCRs, all DNA extracted from animal samples was subjected to PO PCR amplification and showed fragments at the expected 470-bp size (Additional file 6: Fig. S2). This PCR targets a mammalian gene whose amplification would provide information about the DNA quality and possible presence of inhibitors [22].

Using 7SL primers, the conventional PCR method failed to amplify the target *Leishmania* DNA in any of the animal samples, despite the addition of DMSO at various percentages (5% and 10%). However, expected fragments at 280 bp were found using the second pair of primers targeting HSP70 genes (Additional file 5: Fig. S1). The results of PO and conventional 7SL and HSP70 PCRs on the DNA of animal samples are summarized in Table 1.

**Table 1:** Results of the molecular tools applied on samples from the different reservoir specimens

Animals	Specimens code	Samples^a^	Conventional PCR		Sequencing of HSP70 PCR product^c^	Species identification by PCR-HRM^b^		Sequencing of PCR–HRM products^c^	
			PO	HSP70^b^		7SL	HSP70	7SL	HSP70
	EMPA10^d^	NA	+	+	*L. major*	*L. major*	*L. major*	*L. major*	–
	LV50^d^	NA	+	+	*L. infantum*	*L. infantum*	*L. infantum*	*L. infantum*	–
	L75^d^	NA	+	+	*L. tropica*	*L. tropica*	*L. tropica*	–	–
Hedgehogs	ED1	F (3)	+	+	*L. major*	*L. major*	*L. major*	*L. major*	–
		B (4)	+	+	*L. major*	*L. major*	*L. major*	–	*L. major*
		C (3)	+	+	–	*L. infantum*	*L. infantum*	–	–
		R (3)	+	+	–	*L. infantum*	*L. infantum*	–	–
		S (3)	+	+	*L. major*	*L. major*	*L. major*	–	–
		P (2)	+	+	–	*L. infantum*	*L. infantum*	–	–
		LN (2)	+	+	–	*L. infantum*	*L. infantum*	–	–
	ES1	F (3)	+	+	*L. major*	*L. major*	*L. major*	–	–
		R (3)	+	+	–	*L. infantum*	*L. infantum*	–	–
		S (3)	+	+	*L. infantum*	*L. infantum*	*L. infantum*	–	–
		C (3)	+	+	*L. infantum*	*L. infantum*	*L. infantum*	–	–
		P (3)	+	+	–	*L. tropica*	*L. tropica*	–	–
	EZ4	F (2)	+	+	*L. major*	*L. major*	*L. major*	–	–
		R (2)	+	+	–	*L. major*	*L. major*	–	–
		S (3)	+	+	*L. major*	*L. major*	*L. major*	–	–
		C (3)	+	+	–	*L. infantum*	*L. infantum*	–	–
		LN (2)	+	+	*L. major*	*L. major*	*L. major*	–	–
Dogs	10	B (3)	+	+	*L. infantum*	*L. infantum*	*L. infantum*	*L. infantum*	–
	32	B (2)	+	–	–	*L. infantum*	–	*L. infantum*	–
	37	B (2)	+	+	*L. infantum*	*L. infantum*	*L. infantum*	*L. infantum*	–
	43	B (2)	+	+	*L. infantum*	*L. infantum*	–	–	–
	47	B (2)	+	+	–	*L. infantum*	*L. infantum*	*L. infantum*	–
	24	B (2)	+	–	–	–	–	–	–
	5	B (2)	+	–	–	–	–	–	–
	20	B (2)	+	–	–	–	–	–	–
	25	B (2)	+	–	–	–	–	–	–
	46	B (3)	+	–	–	*L. infantum*	*L. infantum*	–	–
	3	B (2)	+	–	–	*L. infantum*	*L. infantum*	–	–
	4	B (3)	+	–	–	*L. infantum*	*L. infantum*	–	–
*Meriones*	MZ1	S (4)	+	+	*L. major*	*L. major*	*L. major*	–	–
		F (3)	+	+	*L. major*	*L. major*	*L. major*	–	–
		R (3)	+	+	–	*L. major*	–	–	–
	MZ2	S (4)	+	+	*L. major*	*L. major*	*L. major*	*L. major*	–
		F (2)	+	+	–	*L. major*	*L. major*	–	–
		R (2)	+	+	–	*L. major*	*L. major*	–	–
	MZ3	S (2)	+	+	*L. infantum*	*L. infantum*	*L. infantum*		
		R (3)	+	+	*L. infantum*	*L. infantum*	*L. infantum*	–	–
		C (2)	+	–	–	*L. major*	*L. major*	–	–
	MZ4	S (2)	+	–	–	–	–	–	–
		F (2)	+	+	*L. major*	*L. major*	*L. major*	–	–
		R (2)	+	+	*L. major*	*L. major*	*L. major*	*L. major*	–
	MZ5	S (2)	+	+	–	*L. major*	*L. major*	–	–
		F (4)	+	+	*L. infantum*	*L. infantum*	*L. infantum*	–	–
		R (2)	+	–	–	–	–	–	–
		C (4)	+	–	–	*L. major*	*L. major*	–	–
	MZ6	S (2)	+	+	*L. infantum*	*L. infantum*	*L. infantum*		
		F (2)	+	+	*L. infantum*	*L. infantum*	*L. infantum*	–	–
		R (2)	+	–	–	*L. major*	*L. major*	–	–
	MZ7	F (3)	+	+	*L. major*	*L. major*	*L. major*	–	–
		S (3)	+	+	*L. infantum*	*L. infantum*	*L. infantum*	–	–
		R (2)	+	–	–	*L. major*	*L. major*	–	–
		C (2)	+	–	–	–	–	–	–

*NA* not applicable, *B* blood, *C* heart, *S* spleen, *R* kidney, *F* liver, *LN* lymph node, *P* peritoneum

^a^ Numbers between parentheses indicate the number of replicates in PCR–HRM reactions

^b^ The (–) sign indicates that no amplification product was obtained

^c^ The (–) sign indicates that no sequencing reaction was done or the sequence was of poor quality and could not be interpreted

^d^ Species used as references in PCR–HRM reactions

#### Sequencing of conventional PCR products

In order to assess the identity of amplified fragments obtained by conventional 7SL and HSP70 PCR from in vitro-cultured *Leishmania* parasites and on animal samples, direct Sanger sequencing was carried out. Thus, amplified products from LV50, EMPA10, and L75 isolates corresponding respectively to *L. infantum*, *L. major*, and *L. tropica* obtained by both pairs of primers (7SL and HSP70) were sequenced in both directions. Obtained sequences were subjected to BLAST searches and showed matches with the three corresponding *Leishmania* species sequences with significant coverage, identity percent and E values (Additional file 7: Table S5). Sequencing results of HSP70 amplified products from samples of the collected *Meriones* showed infection with *L. major* of the liver and spleen of MZ1, spleen of MZ2, liver and kidney of MZ4, and liver of MZ7, while the spleen and kidney of MZ3, liver of MZ5, spleen and liver of MZ6, and spleen of MZ7 were found to be infected by *L. infantum*. Interestingly, the MZ7 specimen showed co-infection with both species *L. infantum* and *L. major* that were found in the spleen and liver samples, respectively (Table 1). For hedgehog specimens, the blood and liver of ED1, liver of ES1, and liver, spleen, and lymph node of EZ4 were found to be infected by *L. major*, while *L. infantum* was detected in the spleen and heart of ES1. Concerning dog specimens, the blood of dog 10, dog 37, and dog 43 was found to be infected by *L. infantum* (Table 1; Additional file 7: Table S5).

#### PCR-HRM assays

**Fig. 1:**
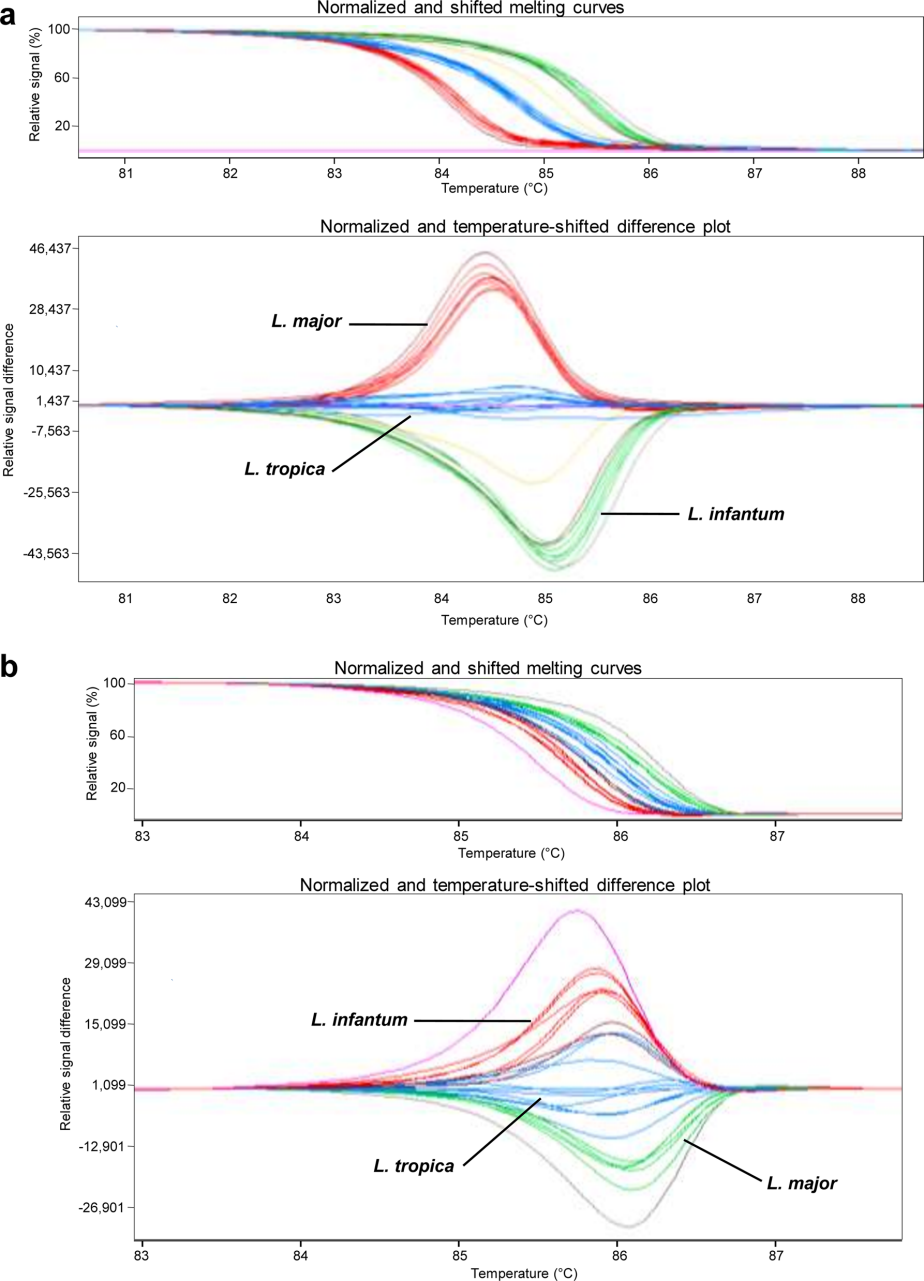
Gene scanning analysis showing discrimination of 27 strains representing the three *Leishmania* species *L. infantum*, *L. major*, and *L. tropica* with PCR-HRM using 7SL RNA (**a**) and HSP70 (**b**) genes. *Leishmania* strains used are *L. major* (*n* = 8; EMPA10, IL32, Ron 44, EMPA11, EMPA12, Ron155, IL53, and FMH); *L. infantum* (*n* = 10; IPT1, LV08, LV10, LV49, LV50, D5, D8, D11, D13, and D16); and *L. tropica* (*n* = 9; K27, LA28, L75, Adhanis, Gabai159, Bumm30, Rachnan, Bag9, and Bag17)

The PCR-HRM reaction setup was established through several optimization steps, which specifically included addition or no addition of DMSO at different concentrations, and the use of touchdown cycling for primer annealing. To obtain optimal conditions, experiments were first carried out using DNA extracted from the three reference species *L. infantum*, *L. major*, and *L. tropica*. Results showed distinguishable melting peaks based on different Tm values (Table 2), with 10% DMSO and a touchdown program for 7SL primers, and 10% DMSO, without touchdown, for HSP70 primers. To ensure reproducibility and specificity of our PCR-HRM assays, a panel of 27 *Leishmania* strains representing the three reference species (10 *L. infantum*, eight *L. major*, and nine *L. tropica*) were then tested using the same conditions, with both primers. Results showed that each reference species exhibited a distinct HRM profile with a specific Tm melting peak. Moreover, tested strains were grouped into three clusters, allowing their clear discrimination according to species identity (Fig. 1).

**Table 2:** Tm values of average melting curve peak for each *Leishmania* species

Species	Tm value range (mean Tm ± SD) (°C)	
	7SL gene	HSP70 gene
*Leishmania major*	73.67 ± 0.20	70.60 ± 0.07
*Leishmania infantum*	72.67 ± 0.35	70.82 ± 0.03
*Leishmania tropica*	73.04 ± 0.41	69.92 ± 0.03

In order to determine the sensitivity of the PCR-HRM targeting 7SL and HSP70 genes, tenfold dilutions of DNA from each reference *Leishmania* isolate, representing *L. infantum* (MHOM/TN/94/LV50), *L. major* (MHOM/TN/2011/EMPA10), and *L. tropica* (MHOM/IQ/65/L75), were used, ranging from 20 ng to 0.1 pg (DNA amount corresponding to 2 × 10^5^ to one parasite [15]). The detection limit was 0.1 pg DNA in *L. tropica* with both 7SL and HSP70 PCR-HRM, while 1 pg was detected in *L. infantum* by 7SL PCR-HRM and 0.1 pg by HSP70 PCR-HRM in the same species. In the case of *L. major*, HSP70 PCR-HRM allowed detection of 0.1 pg of DNA and 7SL PCR-HRM 0.01 ng (Additional file 8: Fig. S3). No fluctuation in melting curves or Tm values was obtained over the DNA concentration range examined for each species by either PCR-HRM tool (data not shown).

### Validation of PCR-HRM tool for *Leishmania* infection identification in reservoir animals

We tested a total of 52 samples taken from seven *Meriones* (*n* = 23), three hedgehogs (*n* = 17), and 12 dogs (*n* = 12) using 7SL and HSP70 primers. The remaining animal samples were not processed by PCR-HRM due to limited resources in relation to the LightCycler^®^ 480 High Resolution Melting kit availability.

**Fig. 2:**
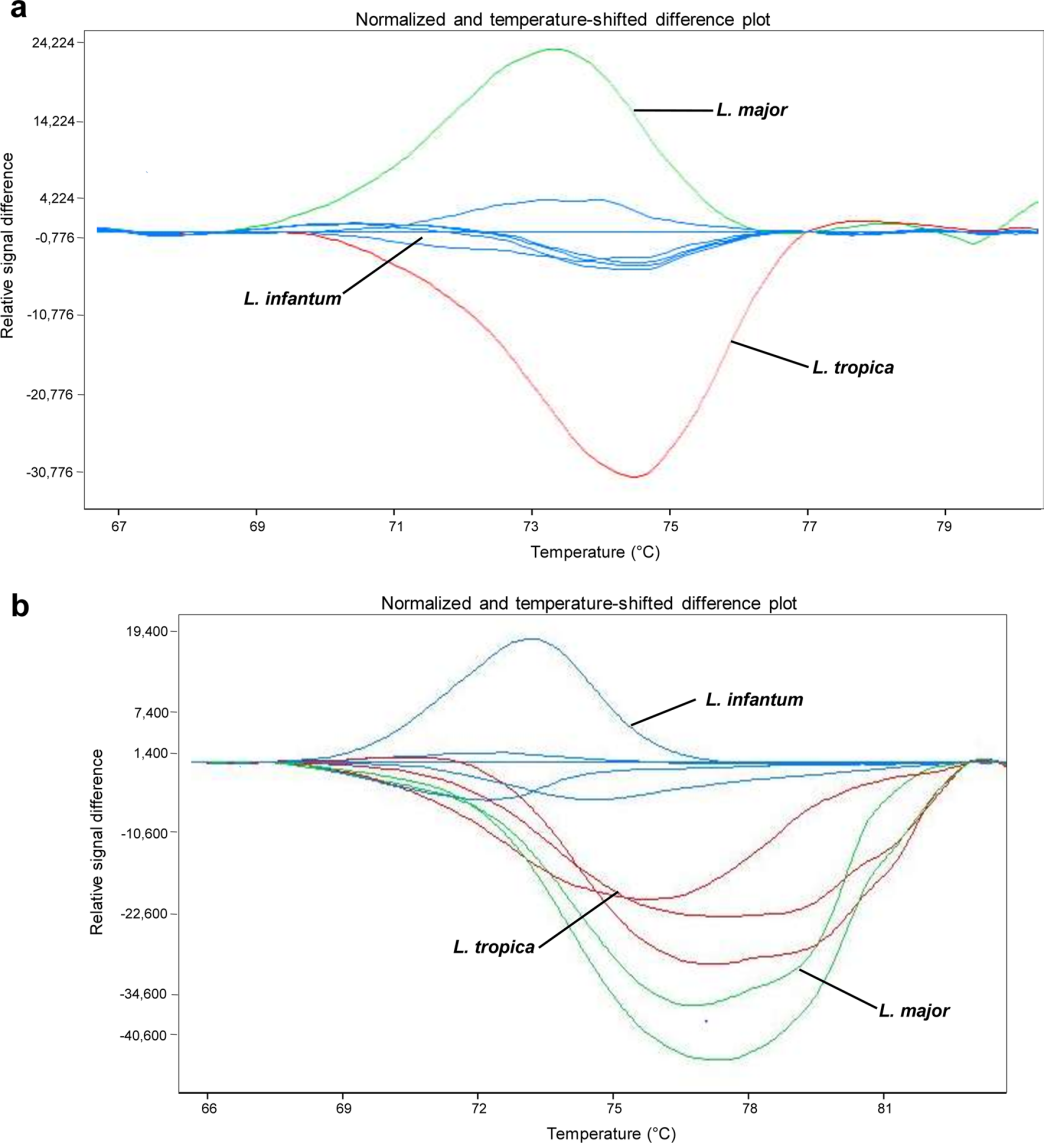
Gene scanning analysis of PCR-HRM from reservoir samples, using 7SL RNA gene. **a** Infection of dogs with *L. infantum*. Tested samples are dog 10, dog 32, dog 37, dog 43, and dog 47. **b** Identification of *Leishmania* species infecting hedgehog, *Meriones*, and dogs. Tested samples are hedgehogs (CEZ4, PES1, and FES1), *Meriones* (SMZ3, SMZ4, and SMZ1), and dogs (dog 3 and dog 4). Reference isolates correspond to *L. infantum*, *L. major*, and *L. tropica*. See Table 1 for an explanation of samples codes

**Fig. 3:**
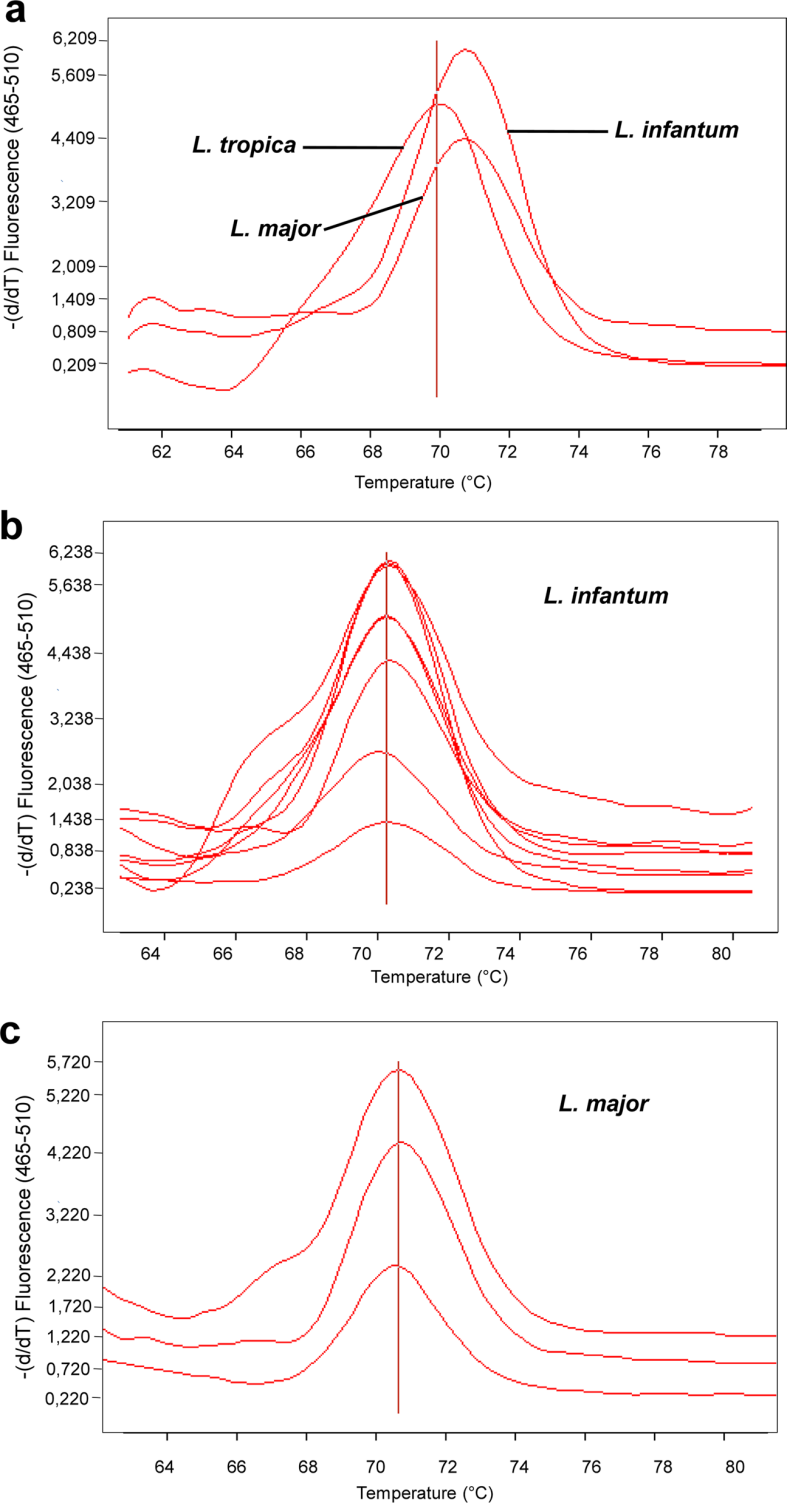
Melting peaks analysis of PCR-HRM from reservoir samples, using HSP70 gene. **a** Melting peaks of reference isolates *L. infantum*, *L. major*, and *L. tropica*. **b** Infection of reservoir samples with *L. infantum*. **c** Infection of reservoir samples with *L. major*. Tested samples are dog 47, CED1, SMZ3, CMZ4, SMZ2, SMZ1, SMZ7, RMZ4, and FMZ1. See Table 1 for explanation of sample codes

The results of the two assays were concordant, and both methods showed that overall among the total samples examined, 45 were positive while seven were negative, with no *Leishmania* infection (Table 1). The results were confirmed by repeating the experiments through two to three independent reactions according to the availability of DNA material (Table 1). Good Ct values were found, providing for each tested sample reliable Tm values that were in accordance with the ranges of the corresponding reference samples, thus allowing us not only to demonstrate the infection of corresponding organs with *Leishmania*, but also to assign them to appropriate species. Moreover, the gene scanning software facilitated samples analysis and allowed a clearer discrimination between species, which was observed through well-separated melting profiles for each *Leishmania* species, revealing distinct clusters. Examples of gene scanning analysis using the 7SL gene and melting peak analysis and using the HSP70 gene are given in Figs. 2 and 3.

This analysis allowed attributing to each infected animal sample a specific *Leishmania* species. Indeed, generally, samples extracted from *Meriones* were found infected with *L. major* while dogs were infected with *L. infantum* (Table 1; Fig. 2). Meanwhile, co-infections with *L. major/L. infantum* were also recorded for *Meriones* specimens MZ3, MZ5, MZ6, and MZ7 (Table 1). However, co-infections with *L. major*/*L. infantum* were found in all tested hedgehogs in different samples of the same specimen (ED1, ES1, and EZ4) (Table 1). Interestingly, infection with the three *Leishmania* species *L. major*, *L. infantum*, and *L. tropica* was found in ES1 hedgehog specimen (Table 1).

Sequence analysis of some of the PCR-HRM products using 7SL and HSP70 targets, corroborated the found *Leishmania* species in analyzed samples, as shown by the BLAST homology search (Table 1; Additional file 7: Table S5).

## Discussion

In the last few years, HRM PCR has become more widely applied for epidemiological purposes related to infectious diseases in addition to genotyping yeasts, viruses, or bacteria [23–26]. Indeed, this technique allows distinction of the different viral or bacterial strains [23–25], detection of mutated and wild-type strains [13], mutations located on resistance genes, such as resistance to rifampicin in *Mycobacterium tuberculosis* [27], or differentiation between *Brucella* species [28]. In parasitology, HRM analysis is extensively applied for protozoan parasites. For example, based on the 18S ribosomal gene, it allowed the rapid diagnosis of bovine babesiosis [29]. It was also evaluated to monitor *Plasmodium falciparum* treatment efficacy [30] and to detect and differentiate among *Theileria annulata*, *Theileria orientalis*, and *Theileria sinensis* species [31]. *Taenia* spp. and filarial worms are the helminths most frequently studied using this assay [32]. For leishmaniasis, HRM was used to identify *L. infantum* in canine serum samples [33]. In addition, it allowed the rapid discriminatory diagnosis of co-infecting *Leptomonas seymouri* with *Leishmania donovani* in clinical samples [34], the discrimination of Old World and New World *Leishmania* species [14–18], and also identification of this parasite in biopsies of patients with a different clinical manifestation of CL in Peru [35].

Tunisia is among the most leishmaniasis-affected countries in the world, with four co-endemic clinical forms. These forms are caused by different *Leishmania* species and hosted by various reservoirs including dogs, rodents (*Psammomys* and *Meriones* genus), gundies (*C. gundi*), weasels (*Mustela nivalis*), and hedgehogs (*A. algirus* and *P. aethiopicus*) [4, 9, 10, 36–39]. In order to explore *Leishmania* infection in confirmed or suspected reservoir animals, several molecular markers and molecular techniques are available. These include mainly amplification by PCR coupled to RFLP and sequencing analyses. Since a low parasite load is expected to be present in reservoirs, especially when they are asymptomatic [21, 40], PCR assays always amplify genes with a high copy number in order to increase the sensitivity of detection. Thus, the kDNA, HSP70, and ITS1 molecular markers are among the most commonly used. Examples include demonstration of canine infection by *L.* (*Viannia*) *guyanensis* in the Brazilian Amazon using PCR and sequencing of HSP70 genes, upon DNA extraction from blood samples [41]. The presence of *Leishmania* parasites in the skin of different species of bats was also evaluated using kDNA PCR and PCR-RFLP analysis of the HSP70 gene in specimens collected in the Pantanal wetlands in Brazil [42] and also using nested PCR targeting the small subunit ribosomal RNA (SSU rRNA) gene in specimens from central western Brazil [43]. Also, PCR targeting ITS1 genes demonstrated *Leishmania* presence in different species of rodents in VL-endemic areas in Ethiopia [44]. Assessment of *L. infantum* infection in equine populations and in cats in visceral leishmaniasis transmission areas in Brazil was also possible with kDNA amplification from blood samples [45] and with ITS1 PCR from conjunctival swabs, respectively [46]. In Morocco, molecular detection of *L. infantum* and *L. tropica* in rodent species from endemic cutaneous leishmaniasis areas was assessed by PCR targeting the SSU rRNA and ITS1 genes, followed by sequencing [47].

Despite the extensive use of PCR and PCR-derived assays, their potential in detecting and identifying *Leishmania* infection, especially in reservoir animals, remains controversial. Indeed, in most cases, re-amplification or nested PCR is necessary to demonstrate *Leishmania* infection, and species identification requires additional steps, mainly RFLP and sequencing. Combining different PCR assays with different gene targets is also used. In addition, these methods are time consuming, especially in post-PCR processing, which increases the risk of DNA contamination. In this context, PCR-HRM methods may constitute a better choice for *Leishmania* infection detection and analysis in reservoir hosts, since they have the advantages of being simpler, less expensive, highly sensitive, and faster than conventional PCR assays. On average, PCR-HRM tests have been calculated to be five times cheaper and three times faster than other types of analysis such as multilocus sequence typing (MLST) and RFLP [48].

In the present study, we investigated *Leishmania* infection in a range of field samples taken from three hedgehogs, seven *Meriones* and 12 dogs captured in the same governorate of El Kef, situated in North West Tunisia, and constituting an active focus of SCL due to *L. infantum*. We used HRM PCR, which is a real-time PCR based on SYBR Green dye, with the Roche LightCycler^®^ 480 machine equipped with gene scanning software (version 1.5.0). Development and optimization of PCR-HRM targeting the 7SL RNA and HSP70 genes required modification of several reaction parameters, including a touchdown protocol, which ultimately improved amplification of the 7SL RNA gene targets. Also, the addition of DMSO enhanced both 7SL RNA and HSP70 PCR reactions. Previous studies have used touchdown PCR protocols to enhance specificity and product formation [18, 49]. This approach offers a simple and rapid means of optimizing PCRs and specifically addresses the limitations inherent in Tm calculations, thus increasing sensitivity, specificity, and product formation [50]. Also, it largely relieves the need for lengthy optimization processes and redesign of primers [51].

The sensitivity of HSP70 PCR-HRM tool reached 0.1 pg of *Leishmania* DNA, equivalent to one parasite, in all tested species, while 7SL PCR-HRM allowed to detect different DNA amounts over the three species. These discrepancies are probably due to the target sequences as well as amplification conditions. Indeed, 0.02 pg DNA were detected using the same primers in 7SL PCR-HRM, in a previous study where in vitro-cultured parasite species from the Old World, were used in different experimental conditions (addition of DMSO and touchdown in the annealing step), as done in our work [17]. In another study targeting the ITS1 rRNA region, 0.05 pg DNA was detected by PCR-HRM from Old World cultured promastigotes [16]. Various DNA amounts, ranging from 50 pg to 500 fg, representing the limit of detection, were also found in a recent study were PCR-HRM targeting the amino acid permease 3 (aap3) gene was tested on different *Leishmania* species from the Old World and the New World [52].

The 7SL RNA and HSP70 PCR-HRM tools used here showed their applicability on samples collected from field animals constituting confirmed or potential reservoirs of *Leishmania* in Tunisia, which brings new information regarding identity/infectivity of reservoir hosts within a specific endemic region. It is worth mentioning here that there are very few publications addressing leishmaniasis diagnosis using HRM in Old World settings. In addition, previously developed PCR-HRM were mostly applied to in vitro-cultured parasites and validated in clinical human samples [15–17], naturally infected sand flies or experimentally infected mice [15, 52], and rarely on reservoir animals [16]. PCR-HRM results on field animals with both 7SL and HSP70 gene targets were nicely concordant and demonstrated co-infection of all tested hedgehogs with *L. major* and *L. infantum*, as shown by differential infection of samples of the same specimen with different *Leishmania* species. However, co-infection in the same sample of a given hedgehog specimen was not found, contrary to what has been previously described [9, 10]. This could be explained by a targeted amplification that results in competitive priming and preferential amplification of sequences corresponding to a specific species [53]. Demonstrating *Leishmania* infection followed by species identification in hedgehogs was previously realized in our laboratory using combined conventional PCR and PCR-RFLP, using different targets. Indeed, amplification of kinetoplast minicircles [54], rRNA gene [55], and nested PCR-RFLP targeting a repetitive intergenic sequence [56, 57] were combined to show natural *Leishmania* infection of hedgehogs in Tunisia [9, 10]. A combination of different PCR assays was necessary, since amplification of parasite DNA from field samples poses challenges with respect to sensitivity and specificity, as parasite load is generally markedly lower in proportion to host DNA [58]. This favors the use of PCR-HRM, which is characterized by very high sensitivity of detection, especially in field sampling. On the other hand, our results further corroborate previous studies that showed natural *Leishmania* infection of hedgehogs and suggest its potential role as a reservoir host, not only in Tunisia [9, 10] but also in Algeria, where it was first described [8], and recently in Iran [7].

Natural *Leishmania* infection of *Meriones* rodents is shown here for the first time by PCR-HRM. Indeed, among the seven studied *M. shawi* specimens, three were found to be infected exclusively by *L. major*, while co-infections with *L. major* and *L. infantum* were also recorded in four of them. Finding these rodents infected with *L. major* is not surprising, since they are considered one of the main reservoir animals of ZCL in both Tunisia and other Mediterranean countries [38, 59, 60]. However, finding *L. infantum* infecting *Meriones* has also been documented in a few studies, in Morocco [47], Iran [61], and China [62]. On the other hand, mixed *Leishmania* species infection of natural reservoirs has rarely been reported. Indeed, within ZCL endemic regions in Iran, mixed infection with *L. major* and *L. turanica* was found in gerbils belonging to the species *Rhombomys opimus* and *Meriones libycus* [63], while co-infection by *L. major* and *L. turanica* or by *L. major* and *L. gerbilli* was described in *R. opimus* rodents [64].

In this study, infection of dogs by *L. infantum* was also demonstrated by 7SL and HSP70 PCR-HRM. Indeed, with both targets, eight out of 12 tested dogs were found to be infected with *L. infantum*. These findings corroborate previous knowledge of the role of dogs as the main reservoir of *L. infantum* causing VL in the MENA [Middle East and North Africa] region [60]. Nonetheless, it is worth noting here that all tested dogs presented with no leishmaniasis symptoms, which further shows the usefulness of these PCR-HRM tools in the diagnosis of canine leishmaniasis (CanL). Indeed, even when they are asymptomatic, dogs are considered carriers and are as infectious as the symptomatic ones [65, 66]. Thus, using sensitive tools like PCR-HRM for CanL diagnosis will enhance the estimation of the real number of infected dogs and better evaluate the extent of infection, thus enhancing the effectiveness of control programs.

## Conclusions

The PCR-HRM assays developed in this study constitute valuable tools for molecular detection and identification of *Leishmania* parasites in reservoir hosts and can be easily implemented in epidemiological surveys in endemic regions, thus providing important insights for future control and prevention strategies for public and animal health.

## Supplementary Information

**Additional file 1: Table S1.** Morphological criteria of the studied *Meriones* and hedgehogs, date of capture, and geographical origins.

**Additional file 2: Table S2.** List of collected samples from the studied dogs, *Meriones*, and hedgehogs.

**Additional file 3: Table S3.** List of *Leishmania* strains used in conventional PCR and PCR-HRM reaction setups.

**Additional file 4: Table S4.**Conventional PCRs primers sequences, reactions, and cycling conditions.

**Additional file 5: Figure S1.** Conventional PCR targeting 7SL RNA and HSP70 genes. **a** Conventional 7SL PCR. Samples from hedgehogs are 1, SED1; 2, FES1. Samples from *Meriones* are 3, FMZ4; 4, SMZ1; 5, SMZ2; 6, GMZ2; 7, SMZ7; 8, FMZ7; 9, RMZ4; 10, FMZ5. Samples from dogs are 11, dog 32. 12, negative (no DNA). **b** Conventional HSP70 PCR. Samples from *Meriones* are 1, SMZ2; 2, GMZ2; 3, SMZ1; 4, SMZ7; 5, FMZ7. 9, negative (no DNA). Reference *Leishmania* DNA are Lt, *L. tropica* (L75); Lm, *L. major* (EMPA10); Li, *L. infantum* (LV50). –, negative (no DNA). M, molecular weight marker 100 bp. All marked sizes are in bp.

**Additional file 6: Figure S2.** PO PCR. Samples correspond to positive controls from mammalian DNA (lanes 1 to 3) and DNA from dogs’ blood (lanes 4 to 9). 1, PBMC (peripheral blood mononuclear cell); 2, ADNh (human DNA); 3, ADNH (hedgehog); 4, dog 10; 5, dog 32; 6, dog 47; 7, dog 49; 8, dog 37; 9, dog 43; –, negative control (no DNA). M, molecular weight marker 50 bp. All marked sizes are in bp.

**Additional file 7: Table S5.** BLAST analysis confirming *Leishmania* identity in a selection of sequences generated upon direct sequencing of conventional HSP70 PCR products and PCR-HRM products of 7SL and HSP70 targets.

**Additional file 8: Figure S3.** Sensitivity of PCR-HRM targeting 7SL RNA and HSP70 genes. Detection limit of PCR-HRM using 7SL RNA and HSP70 genes was determined using tenfold dilutions of DNA from each reference *Leishmania* isolate, representing *L. infantum* (MHOM/TN/94/LV50), *L. major* (MHOM/TN/2011/EMPA10), and *L. tropica* (MHOM/IQ/65/L75). **a.** 7SL PCR-HRM using *L. infantum* DNA **b.** HSP70 PCR-HRM using *L. infantum* DNA. **c.** 7SL PCR-HRM using *L. major* DNA **d.** HSP70 PCR-HRM using *L. major* DNA. **e.** 7SL PCR-HRM using *L. tropica* DNA **f.** HSP70 PCR-HRM using *L. tropica* DNA. The right sections of the figures show the tested samples, which are color-coded according to the amplification result, red when positive and green when negative. In all panels, sample codes correspond to the following DNA amounts: A: 20 ng; B: 10 ng; C: 1 ng; D: 0.1 ng; E: 0.01 ng; F: 0.001 ng; G: 0.0001 ng; H: negative control (no DNA).

## Data Availability

Not applicable.

## Abbreviations

7SL RNA: 7 Spliced leader RNA gene
CanL: Canine leishmaniasis
CCL: Chronic cutaneous leishmaniasis
CL: Cutaneous leishmaniasis
Ct: Threshold cycle
DMSO: Dimethyl sulfoxide
HSP70: Heat shock protein 70 gene
ITS1: Ribosomal internal transcribed spacer 1
kDNA: Kinetoplast DNA
MLST: Multilocus sequence typing
PCR-HRM: PCR high-resolution melting analysis
PO: Acidic ribosomal phosphoprotein
RFLP: Restriction fragment length polymorphism
SCL: Sporadic cutaneous leishmaniasis
SNP: Single nucleotide polymorphism
SSU rRNA: Small subunit ribosomal RNA genes
Tm: Melting temperature
VL: Visceral leishmaniasis
ZCL: Zoonotic cutaneous leishmaniasis
