# Supplementary material for: High-resolution melting analysis identifies reservoir hosts of zoonotic Leishmania parasites in Tunisia
Source: Parasit Vectors. 2022 Jan 8;15:12. doi: 10.1186/s13071-021-05138-x (PMC8742351; doi:10.1186/s13071-021-05138-x)
Supplement: Supplementary file 1 — Additional file 1: Table S1. Morphological criteria of the studied Meriones and hedgehogs, date of capture, and geographical origins. [file 13071_2021_5138_MOESM1_ESM.docx]

**Table S1** Morphological criteria of the studied *Meriones* and hedgehogs, date of capture and geographical origins.

| Animals | Governorate (Locality) | Specimen code | Date of capture | Sex | Body (cm) | Tail (cm) | Hint Foot (cm) | Ear (cm) | Weight (g) | Species identification | Observations |
| --- | --- | --- | --- | --- | --- | --- | --- | --- | --- | --- | --- |
| *Meriones* | El Kef (Zaafran, Oued Souani) | MZ1 | January 2015 | Male | 14,5 | 14 | 4 | 1,5 | – | *Meriones shawi* | Presence of a lesion on the tail |
|  | El Kef (Zaafran, Oued Souani) | MZ2 | January 2015 | Female | 15,5 | 15,5 | 3,5 | 2,2 | – | *Meriones shawi* | None |
|  | El Kef (Zaafran, Oued Souani) | MZ3 | January 2015 | Male | 14,5 | 13 | 3,5 | 1,5 | – | *Meriones shawi* | None |
|  | El Kef (Zaafran, Oued Souani) | MZ4 | January 2015 | Female | 14,5 | 14 | 3,5 | 1,8 | – | *Meriones shawi* | None |
|  | El Kef (Zaafran, Oued Souani) | MZ5 | January 2015 | Male | 14,5 | 13 | 4 | 2 | 86,6 | *Meriones shawi* | None |
|  | El Kef (Zaafran, Oued Souani) | MZ6 | January 2015 | Female | 12,5 | 12,5 | 3,5 | 2 | 63,1 | *Meriones shawi* | None |
|  | El Kef (Zaafran, Oued Souani) | MZ7 | January 2015 | Male | 16,5 | 14,5 | 4 | 2 | – | *Meriones shawi* | Presence of a lesion on the left ear |
| Hedgehogs | El Kef (Dahmani) | ED1 | July 2019 | Male | 28,2 | 3 | 4,5 | 2,5 | 559, 5 | *Atelerix algirus* | None |
|  | El Kef (Kalet Snen) | ES1 | October 2019 | Male | 18 | 1,5 | 3,5 | 1,7 | 256,5 | *Atelerix algirus* | None |
|  | El Kef (Zaafran, Oued Souani) | EZ4 | October 2019 | Male | 21,5 | 3 | 4 | 3 | 520 | *Atelerix algirus* | None |
