# Supplementary material for: High-resolution melting analysis identifies reservoir hosts of zoonotic Leishmania parasites in Tunisia
Source: Parasit Vectors. 2022 Jan 8;15:12. doi: 10.1186/s13071-021-05138-x (PMC8742351; doi:10.1186/s13071-021-05138-x)
Supplement: Supplementary file 2 — Additional file 2: Table S2. List of collected samples from the studied dogs, Meriones, and hedgehogs. [file 13071_2021_5138_MOESM2_ESM.docx]

**Table S2** List of collected samples from the studied dogs, meriones and hedgehogs.

| Animals | Specimens | Biological samples ^a^ | | | | | | | | | | | | | | Total |
| --- | --- | --- | --- | --- | --- | --- | --- | --- | --- | --- | --- | --- | --- | --- | --- | --- |
| Dogs ^b^ |  | B | – | – | – | – | – | – | – | – | – | – | – | ES | GS | 95 |
| Meriones | MZ1 | – | C | S | R | F | ST | U | – | – | – | – | – | ES | – | 7 |
|  | MZ2 | – | C | S | R | F | – | – | LN | BM | – | – | – | ES | – | 7 |
|  | MZ3 | – | C | S | R | F | – | U | LN | – | – | – | – | ES | – | 7 |
|  | MZ4 | – | – | S | R | F | – | – | LN | – | – | – | – | ES | – | 5 |
|  | MZ5 | – | C | S | R | F | – | U | – | BM | – | – | – | – | – | 6 |
|  | MZ6 | – | C | S | R | F | – | – | – | BM | – | – | – | – | – | 5 |
|  | MZ7 | – | C | S | R | F | – | U | LN | BM | – | – | – | ES | – | 8 |
| Hedgehogs | ED1 | B | C | S | R | F | SE | U | – | BM | P | F | N | ES | GS | 14 |
|  | ES1 | B | C | S | R | F | SE | U | LN | BM | P | – | – | ES | – | 11 |
|  | EZ4 | B | C | S | R | F | – | – | LN | – | – | – | – | – | – | 6 |

*B* Blood, *C* Heart, *S* Spleen, *R* Kidney, *F* Liver, *SE* Skin of ear, *ST* Skin of tail, *U* Urine, *LN* Lymph Node, *BM* Bone marrow, *P* Peritoneum, *F* Fat, *N* Nodule, *ES* Eye swab, *GS* Gum swab.

^a^ Abbreviations indicate the types of samples that were collected from studied dogs, meriones and hedgehogs’ specimens and a – sign indicates that no samples were taken.

^b^ Samples collected from dogs correspond to Blood (43) and ES/GS (52).
