# Supplementary material for: High-resolution melting analysis identifies reservoir hosts of zoonotic Leishmania parasites in Tunisia
Source: Parasit Vectors. 2022 Jan 8;15:12. doi: 10.1186/s13071-021-05138-x (PMC8742351; doi:10.1186/s13071-021-05138-x)
Supplement: Supplementary file 3 — Additional file 3: Table S3. List of Leishmania strains used in conventional PCR and PCR-HRM reaction setups. [file 13071_2021_5138_MOESM3_ESM.docx]

**Table S3** List of *Leishmania* strains used in conventional PCR and PCR–HRM reactions set up.

| International code^a^ | Laboratory code | Zymodeme | Pathology | Species |
| --- | --- | --- | --- | --- |
| MHOM/TN/96/Drep05 | Drep05 | MON-1 | CL | *L. infantum* |
| MHOM/TN/94/Drep08 | Drep08 | MON-1 | CL | *L. infantum* |
| MHOM/TN/97/Drep11 | Drep11 | MON-24 | CL | *L. infantum* |
| MHOM/TN/97/Drep13 | Drep13 | MON-24 | CL | *L. infantum* |
| MHOM/TN/98/Drep16 | Drep16 | MON-24 | CL | *L. infantum* |
| MHOM/TN/92/LV08 | LV08 | – | VL | *L. infantum* |
| MHOM/TN/94/LV10 | LV10 | MON-80 | VL | *L. infantum* |
| MHOM/TN/94/LV49 | LV49 | MON-24 | VL | *L. infantum* |
| MHOM/TN/94/LV50 | LV50 | MON-1 | VL | *L. infantum* |
| MHOM/TN/80/IPT1 * | IPT1 | MON-1 | VL | *L. infantum* |
| MHOM/TN/2011/EMPA10 | EMPA10 | – | CL | *L. major* |
| MHOM/TN/2011/EMPA11 | EMPA11 | – | CL | *L. major* |
| MHOM/TN/2011/EMPA12 | EMPA12 | – | CL | *L. major* |
| MHOM/IL/83/IL53 | IL53 | MON-67 | CL | *L. major* |
| MPSA/TN/87/Ron44 | Ron 44 | MON-25 | NA | *L. major* |
| MPSA/TN/87/Ron155 | Ron 155 | MON-25 | NA | *L. major* |
| MHOM/IL/83/IL32 | IL32 | MON-68 | CL | *L. major* |
| MHOM/TN/90/FMH | FMH | – | CL | *L. major* |
| MHOM/GR/00/LA28 | LA28 | LON-65 | CL | *L. tropica* |
| MHOM/IQ/65/L75 | L75 | MON-6 | CL | *L. tropica* |
| MHOM/IQ//73/Bumm30 | Bumm30 | LON-17 | CL | *L. tropica* |
| MRAT/IQ/73/Adhanis | Adhanis | MON-5 | CL | *L. tropica* |
| MHOM/IL/78/Rachnan | Rachnan | MON-60 | CL | *L. tropica* |
| MHOM/SU/74/SAF-K27 * | K27 | MON-60 | CL | *L. tropica* |
| MHOM/IQ/76/Bag9 | Bag9 | MON-53 | CL | *L. tropica* |
| MHOM/IQ/76/Bag17 | Bag17 | LON-24 | CL | *L. tropica* |
| MHOM/IL/00/Gabai159 | Gabai159 | LON-9 | CL | *L. tropica* |

*VL* visceral leishmaniasis, *CL* cutaneous leishmaniasis, *NA* not applicable, *–* not typed.

^a^ WHO code (host/country/year/code) of the strains.

* WHO reference stocks.
