# Supplementary material for: High-resolution melting analysis identifies reservoir hosts of zoonotic Leishmania parasites in Tunisia
Source: Parasit Vectors. 2022 Jan 8;15:12. doi: 10.1186/s13071-021-05138-x (PMC8742351; doi:10.1186/s13071-021-05138-x)
Supplement: Supplementary file 4 — Additional file 4: Table S4.Conventional PCRs primers sequences, reactions, and cycling conditions. [file 13071_2021_5138_MOESM4_ESM.docx]

| PCR name | Target gene | Primers sequences | Expected fragment size (bp) | Reaction conditions | Cycling conditions | References |
| --- | --- | --- | --- | --- | --- | --- |
| 7SL | Ribosomal RNA subunit | CJ7SLF : 5’ ACGTGGACCAGCGAGGGT 3’  QRT7SLR : 5’ CGGTTCCCTCGCTTCAAC 3’ | 119 | 1X PCR buffer; 0.5 µM each primer; 0.2 mM dNTPs; 1.5 mM MgCl_2_; 10% DMSO; 1.25 units Go Taq DNA polymerase (Promega, France); 50 ng template DNA. | 94°C: 5 min; 35 cycles of: 94°C: 1 min, 61°C: 30 sec, 72°C: 1 min; 72 °C: 10 min. | [17] |
| HSP70 | Heat shock protein 70 | F_Hsp70 : 5’ GAAGATGAAGGAGACGGC 3’  R_Hsp70 : 5’ GCCTTCACCTCGAACACG 3’ | 280 | 1X PCR buffer; 0.5 µM each primer; 0.2 mM dNTPs; 1.5 mM MgCl_2_; 1.25 units Go Taq DNA polymerase (Promega, France); 20 ng template DNA | 94°C: 5 min; 35 cycles of: 94°C: 1 min, 61°C: 30 sec, 72°C: 30 sec; 72°C: 10 min | This work |
| PO | Acidic ribosomal phosphoprotein | POF : 5’ TCATTGTGGGACAGACA 3’  POR : 5’ GGAGAAGGGGGAGATGTT 3’ | 470 | 1X PCR buffer; 0.6 µM each primer; 0.2 mM dNTPs; 1.5 mM MgCl_2_; 1.25 units Go Taq DNA polymerase (Promega, France); 20 ng template DNA. | 94°C: 5 min; 35 cycles of: 94°C: 30 sec, 51°C: 30 sec, 72°C: 1 min; 72°C: 10 min | [22] |

**Table S4** Conventional PCRs primers sequences, reactions and cycling conditions.

*DMSO* Dimethyl Sulfoxide.
