# Supplementary material for: High-resolution melting analysis identifies reservoir hosts of zoonotic Leishmania parasites in Tunisia
Source: Parasit Vectors. 2022 Jan 8;15:12. doi: 10.1186/s13071-021-05138-x (PMC8742351; doi:10.1186/s13071-021-05138-x)
Supplement: Supplementary file 5 — Additional file 5: Figure S1. Conventional PCR targeting 7SL RNA and HSP70 genes. a Conventional 7SL PCR. Samples from hedgehogs are 1, SED1; 2, FES1. Samples from Meriones are 3, FMZ4; 4, SMZ1; 5, SMZ2; 6, GMZ2; 7, SMZ7; 8, FMZ7; 9, RMZ4; 10, FMZ5. Samples from dogs are 11, dog 32. 12, negative (no DNA). b Conventional HSP70 PCR. Samples from Meriones are 1, SMZ2; 2, GMZ2; 3, SMZ1; 4, SMZ7; 5, FMZ7. 9, negative (no DNA). Reference Leishmania DNA are Lt, L. tropica (L75); Lm, L. major (EMPA10); Li, L. infantum (LV50). –, negative (no DNA). M, molecular weight marker 100 bp. All marked sizes are in bp. [file 13071_2021_5138_MOESM5_ESM.pdf]

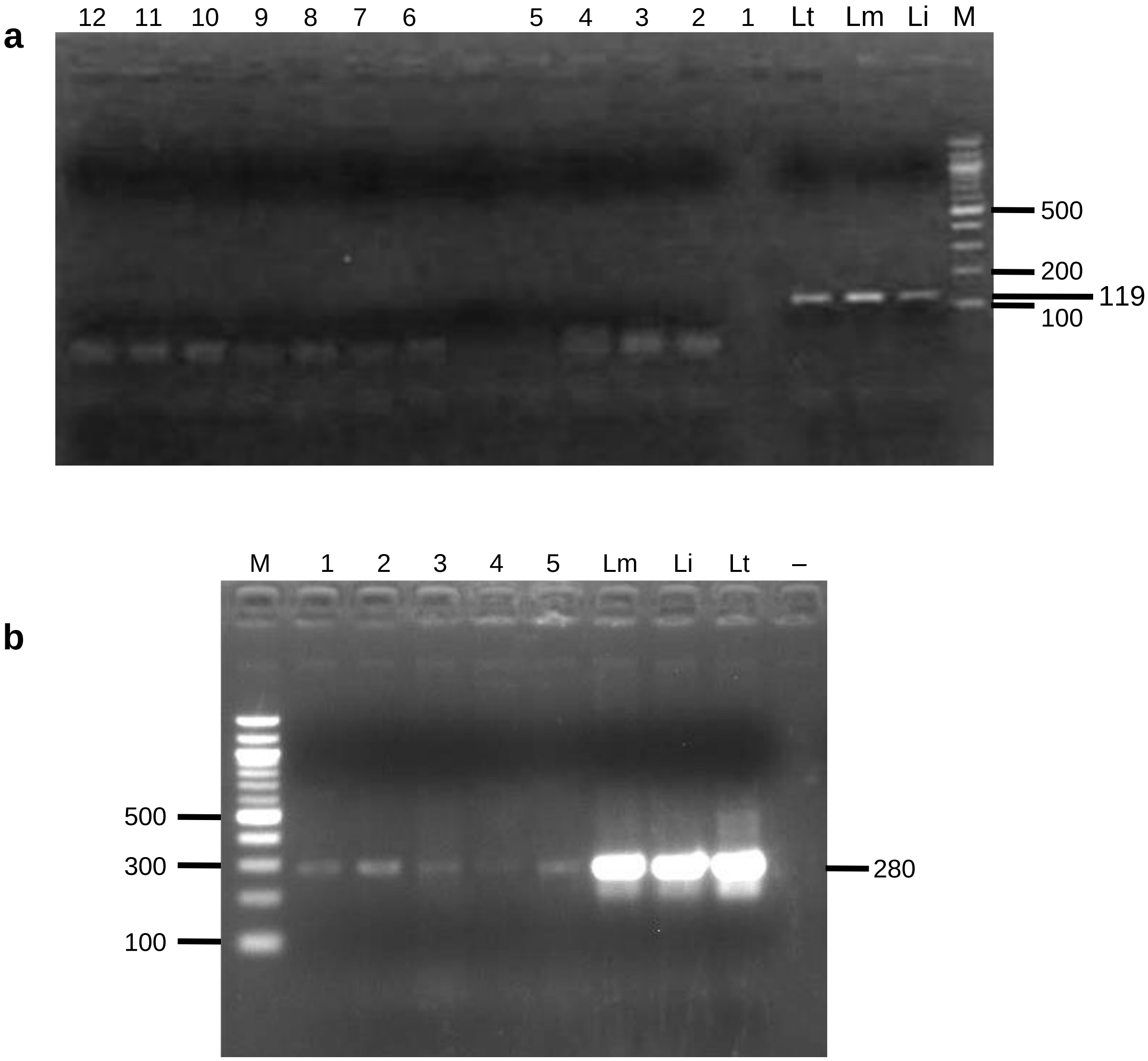

Additional file 5: Figure S1. Conventional PCR targeting 7SL and HSP70 genes.
a Conventional 7SL PCR. Samples from hedgehogs are 1, SED1; 2, FES1. Samples from *Meriones* are 3, FMZ4; 4, SMZ1; 5, SMZ2; 6, GMZ2; 7, SMZ7; 8, FMZ7; 9, RMZ4; 10, FMZ5. Samples from dogs are 11, dog 32. 12, Negative (no DNA).
b Conventional HSP70 PCR. Samples from *Meriones* are 1, SMZ2; 2, GMZ2; 3, SMZ1; 4, SMZ7; 5, FMZ7. 9, Negative (no DNA). Reference *Leishmania* DNA are Lt, *L. tropica* (L75); Lm, *L. major* (EMPA10); Li, *L. infantum* (LV50). – , Negative (no DNA). M, Molecular weight marker 100bp. All marked sizes are in bp.
