# Supplementary material for: High-resolution melting analysis identifies reservoir hosts of zoonotic Leishmania parasites in Tunisia
Source: Parasit Vectors. 2022 Jan 8;15:12. doi: 10.1186/s13071-021-05138-x (PMC8742351; doi:10.1186/s13071-021-05138-x)
Supplement: Supplementary file 6 — Additional file 6: Figure S2. PO PCR. Samples correspond to positive controls from mammalian DNA (lanes 1 to 3) and DNA from dogs’ blood (lanes 4 to 9). 1, PBMC (peripheral blood mononuclear cell); 2, ADNh (human DNA); 3, ADNH (hedgehog); 4, dog 10; 5, dog 32; 6, dog 47; 7, dog 49; 8, dog 37; 9, dog 43; –, negative control (no DNA). M, molecular weight marker 50 bp. All marked sizes are in bp. [file 13071_2021_5138_MOESM6_ESM.pdf]

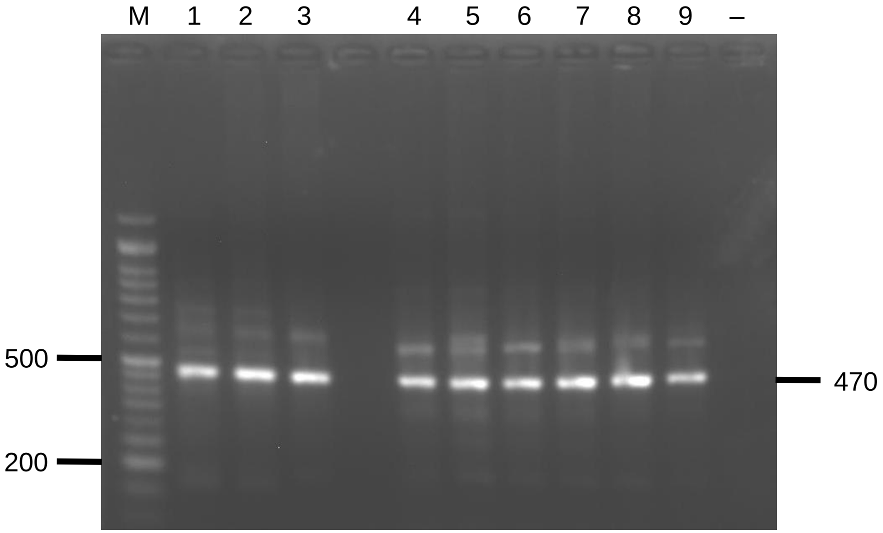

Additional file 6: Figure S2. PO PCR. Samples correspond to positive controls from mammalian DNA (lanes 1 to 3) and DNAs from dogs' blood (lanes 4 to 9). 1, PBMC (peripheral blood mononuclear cell); 2, ADNh (human DNA); 3, ADNH (Hedgehog); 4, dog 10; 5, dog 32; 6, dog 47; 7, dog 49; 8, dog 37; 9, dog 43; – , negative control (no DNA). M, Molecular weight marker 50bp. All marked sizes are in bp.
