# Supplementary material for: High-resolution melting analysis identifies reservoir hosts of zoonotic Leishmania parasites in Tunisia
Source: Parasit Vectors. 2022 Jan 8;15:12. doi: 10.1186/s13071-021-05138-x (PMC8742351; doi:10.1186/s13071-021-05138-x)
Supplement: Supplementary file 7 — Additional file 7: Table S5. BLAST analysis confirming Leishmania identity in a selection of sequences generated upon direct sequencing of conventional HSP70 PCR products and PCR-HRM products of 7SL and HSP70 targets. [file 13071_2021_5138_MOESM7_ESM.docx]

**Table S5** Blast analysis confirming *Leishmania* identity in a selection of sequences generated upon direct sequencing of conventional HSP70 PCR products and of PCR–HRM products of 7SL and HSP70 targets.

|  |  |  | *Leishmania* species | Query Cover | E-value | Percentage of Identity | Accession Number |
| --- | --- | --- | --- | --- | --- | --- | --- |
| Conventional PCR  7SL gene | Reference isolates | LV50 | *L. infantum* | 98% | 7e-32 | 100% | XR_001203134.1 |
|  |  | EMPA10 | *L. major* | 100% | 7e-32 | 100% | FJ592187.1 |
|  |  | L75 | *L. tropica* | 91% | 1e-29 | 98,73% | FJ592182.1 |
| Conventional PCR  HSP70 gene | Reference isolates | LV50 | *L. infantum* | 95% | 4e-110 | 99% | XM_001467062.1 |
|  |  | EMPA10 | *L. major* | 100% | 2e-117 | 100 % | XM_001684806.1 |
|  |  | L75 | *L. tropica* | 95% | 2e-43 | 76 % | Y08020.1 |
|  | Hedgehogs | FED1 | *L. major* | 100% | 4e-100 | 100% | XM_001684806.1 |
|  |  | BED1 | *L. major* | 100% | 4e-95 | 100% | XM_001684806.1 |
|  |  | SED1 | *L. major* | 99% | 3e-101 | 100% | XM_001684806.1 |
|  |  | FES1 | *L. major* | 97% | 3e-101 | 100% | XM_001684806.1 |
|  |  | SES1 | *L. infantum* | 100% | 3e-86 | 100% | XM_001467062.1 |
|  |  | CES1 | *L. infantum* | 100% | 4e-100 | 100% | XM_001467062.1 |
|  |  | FEZ4 | *L. major* | 100% | 8e-87 | 100% | XM_001684806.1 |
|  |  | SEZ4 | *L. major* | 100% | 8e-87 | 100% | XM_001684806.1 |
|  |  | GSEZ4 | *L. major* | 98% | 8e-87 | 100% | XM_001684806.1 |
|  | *Meriones* | SMZ1 | *L. major* | 100% | 1e-105 | 100% | XM_001684806.1 |
|  |  | FMZ1 | *L. major* | 100% | 1e-105 | 100% | XM_001684806.1 |
|  |  | SMZ2 | *L. major* | 100% | 2e-62 | 100% | XM_001684806.1 |
|  |  | SMZ3 | *L. infantum* | 100% | 2e-67 | 100% | XM_001467062.1 |
|  |  | RMZ3 | *L. infantum* | 100% | 2e-87 | 100% | XM_001467062.1 |
|  |  | FMZ4 | *L. major* | 100% | 6e-101 | 100% | XM_001684806.1 |
|  |  | RMZ4 | *L. major* | 100% | 6e-104 | 100% | XM_001684806.1 |
|  |  | FMZ5 | *L. infantum* | 100% | 3e-101 | 100% | XM_001467062.1 |
|  |  | SMZ6 | *L. infantum* | 100% | 3e-107 | 100% | XM_001467062.1 |
|  |  | FMZ6 | *L. infantum* | 100% | 1e-105 | 100% | XM_001467062.1 |
|  |  | FMZ7 | *L. major* | 100% | 6e-101 | 100% | XM_001684806.1 |
|  |  | SMZ7 | *L. infantum* | 100% | 7e-108 | 100% | XM_001467062.1 |

**Table S5 (continued)** Blast analysis confirming *Leishmania* identity in a selection of sequences generated upon direct sequencing of conventional HSP70 PCR products and of PCR–HRM products of 7SL and HSP70 targets.

|  |  |  | *Leishmania* species | Query Cover | E-value | Percentage of Identity | Accession Number |
| --- | --- | --- | --- | --- | --- | --- | --- |
| Conventional PCR  HSP70 gene | Dogs | 10 | *L. infantum* | 100% | 1e-84 | 100% | XM_001467062.1 |
|  |  | 37 | *L. infantum* | 100% | 1e-110 | 100% | XM_001467062.1 |
|  |  | 43 | *L. infantum* | 100% | 9e-102 | 100% | XM_001467062.1 |
| PCR-HRM 7SL gene | Reference isolates | EMPA10 | *L. major* | 100% | 7e-32 | 100 % | [FJ592187.1](https://www.ncbi.nlm.nih.gov/nucleotide/FJ592187.1?report=genbank&log$=nucltop&blast_rank=2&RID=35FDMTP8013) |
|  |  | LV50 | *L. infantum* | 98% | 7e-32 | 100 % | XR_001203134.1 |
|  | Hedgehogs | FED1 | *L. major* | 92% | 6e-09 | 90% | [FJ592187.1](https://www.ncbi.nlm.nih.gov/nucleotide/FJ592187.1?report=genbank&log$=nucltop&blast_rank=2&RID=35FDMTP8013) |
|  | *Meriones* | SMZ2 | *L. major* | 92% | 2e-09 | 92% | [FJ592187.1](https://www.ncbi.nlm.nih.gov/nucleotide/FJ592187.1?report=genbank&log$=nucltop&blast_rank=2&RID=35FDMTP8013) |
|  |  | RMZ4 | *L. major* | 96% | 2e-19 | 93% | [FJ592187.1](https://www.ncbi.nlm.nih.gov/nucleotide/FJ592187.1?report=genbank&log$=nucltop&blast_rank=2&RID=35FDMTP8013) |
|  | Dogs | DOG 10 | *L. infantum* | 97% | 7e-32 | 100% | XR_001203134.1 |
|  |  | DOG 32 | *L. infantum* | 96% | 2e-19 | 92% | XR_001203134.1 |
|  |  | DOG 37 | *L. infantum* | 96% | 5e-30 | 100% | XR_001203134.1 |
|  |  | DOG 47 | *L. infantum* | 98% | 3e-88 | 100% | XR_001203134.1 |
| PCR-HRM HSP70 gene | Hedgehogs | BED1 | *L. major* | 100% | 1e-100 | 100% | XM_001684806.1 |
