# Supplementary figures and images for: High-resolution melting analysis identifies reservoir hosts of zoonotic Leishmania parasites in Tunisia
Source: Parasit Vectors. 2022 Jan 8;15:12. doi: 10.1186/s13071-021-05138-x (PMC8742351; doi:10.1186/s13071-021-05138-x)

**b**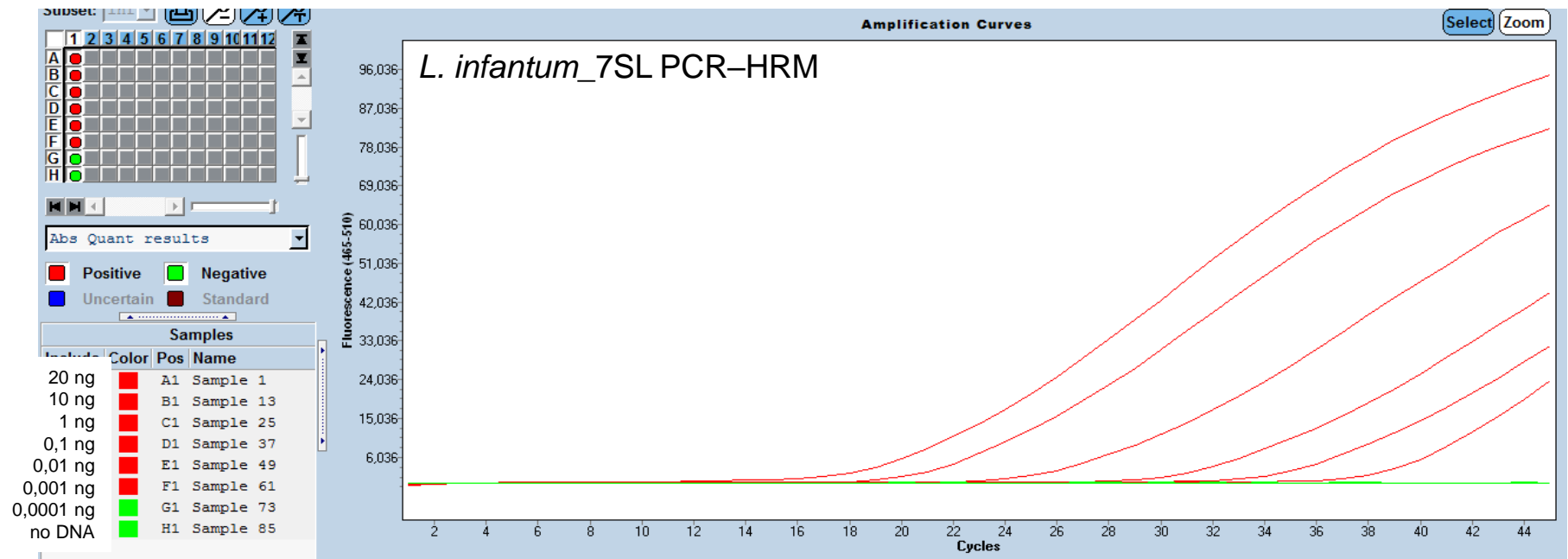**b**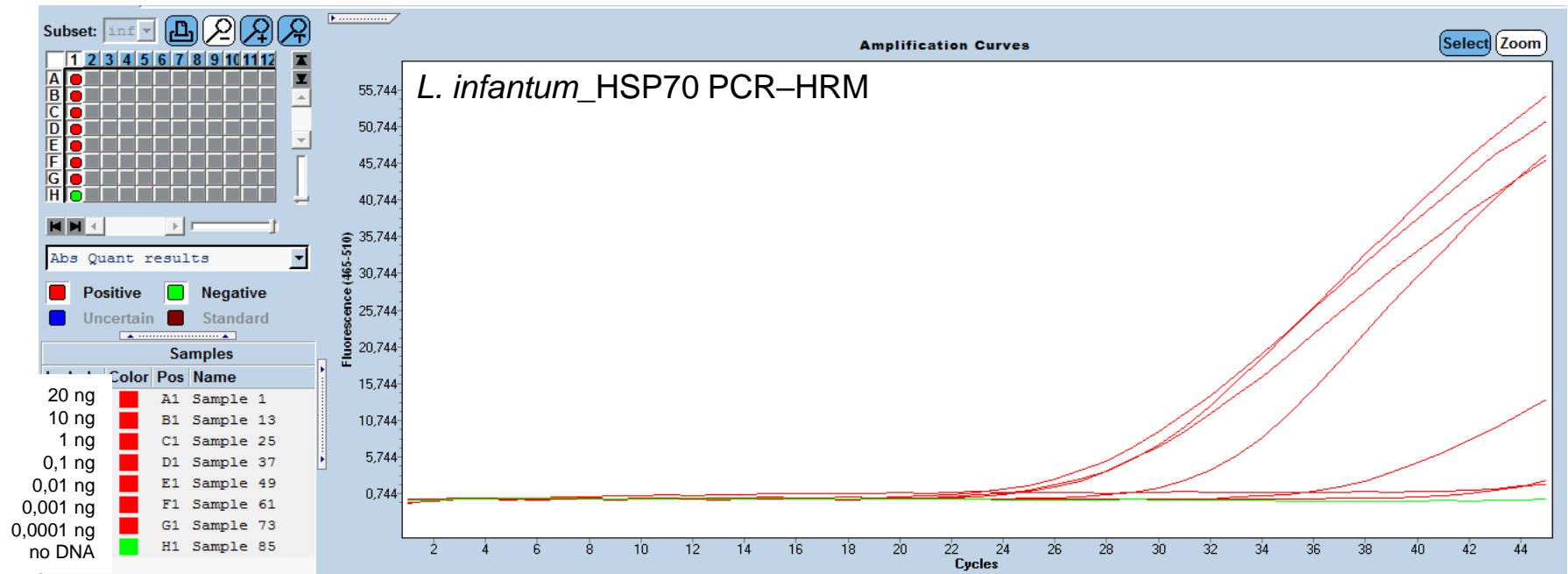

c

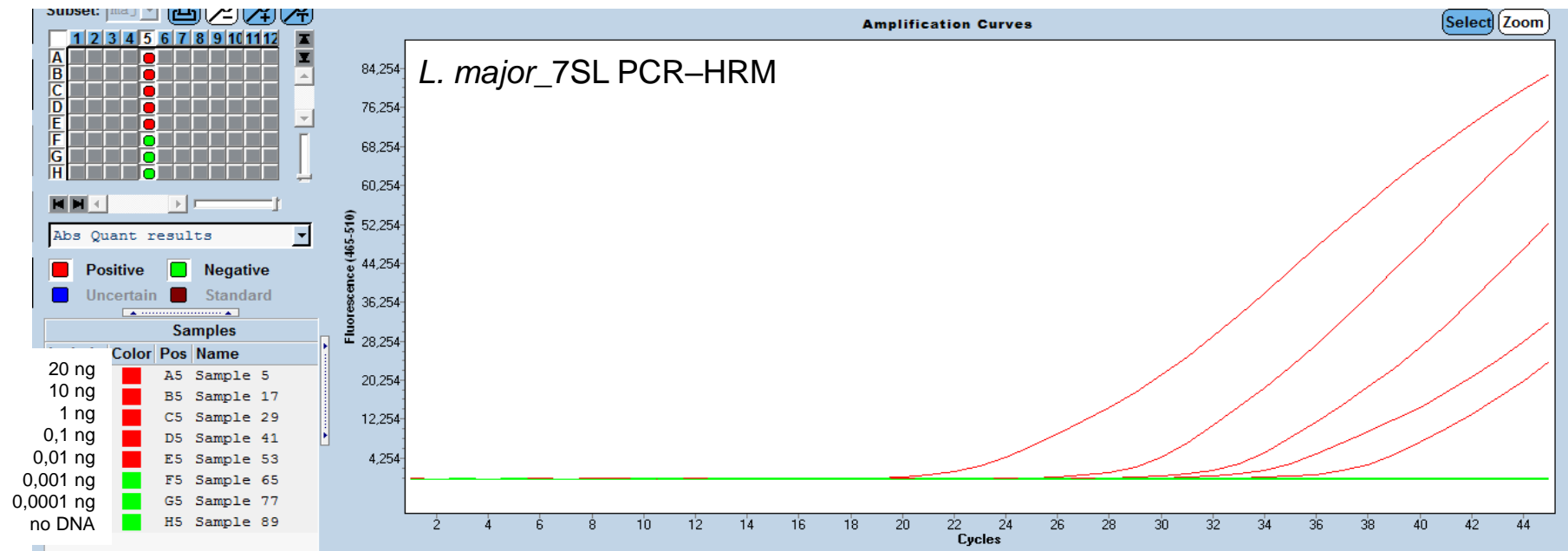

d

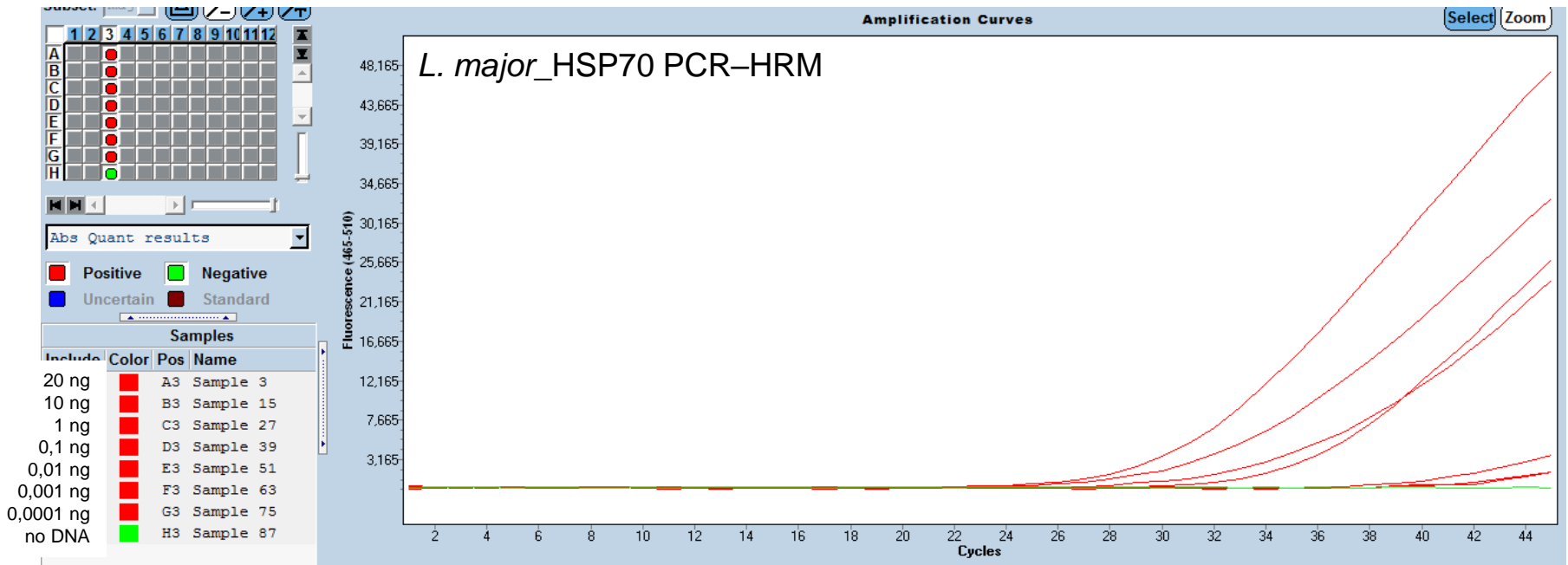

e

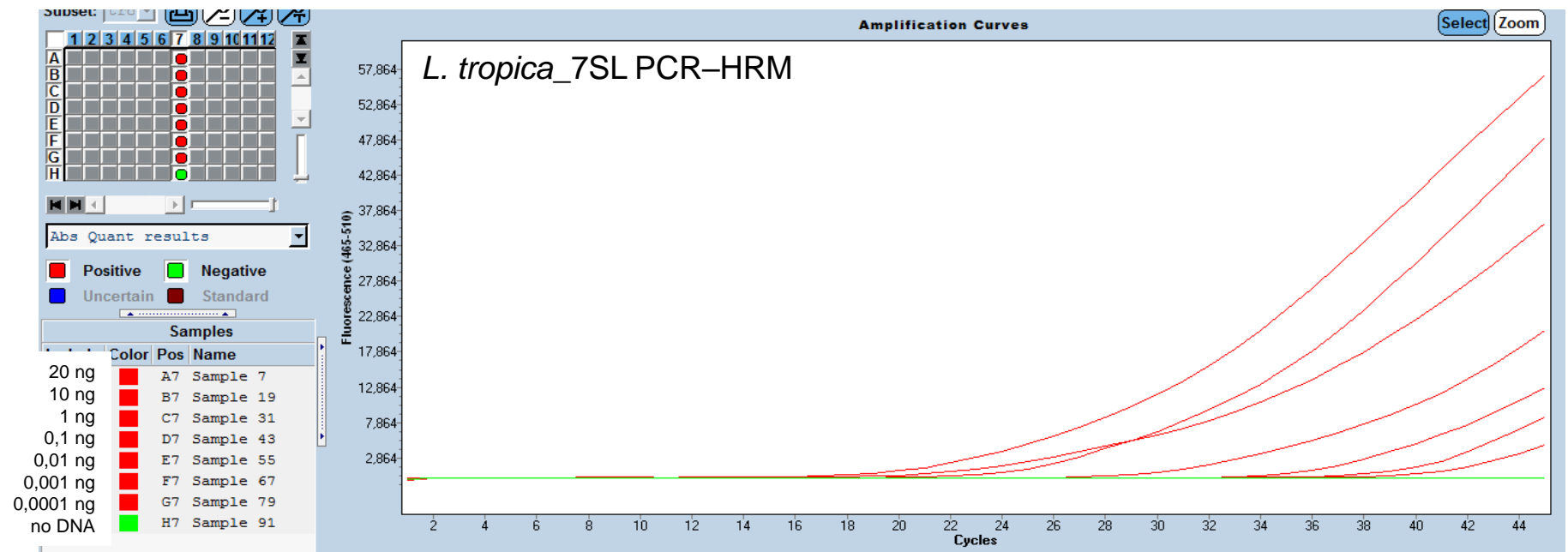

f

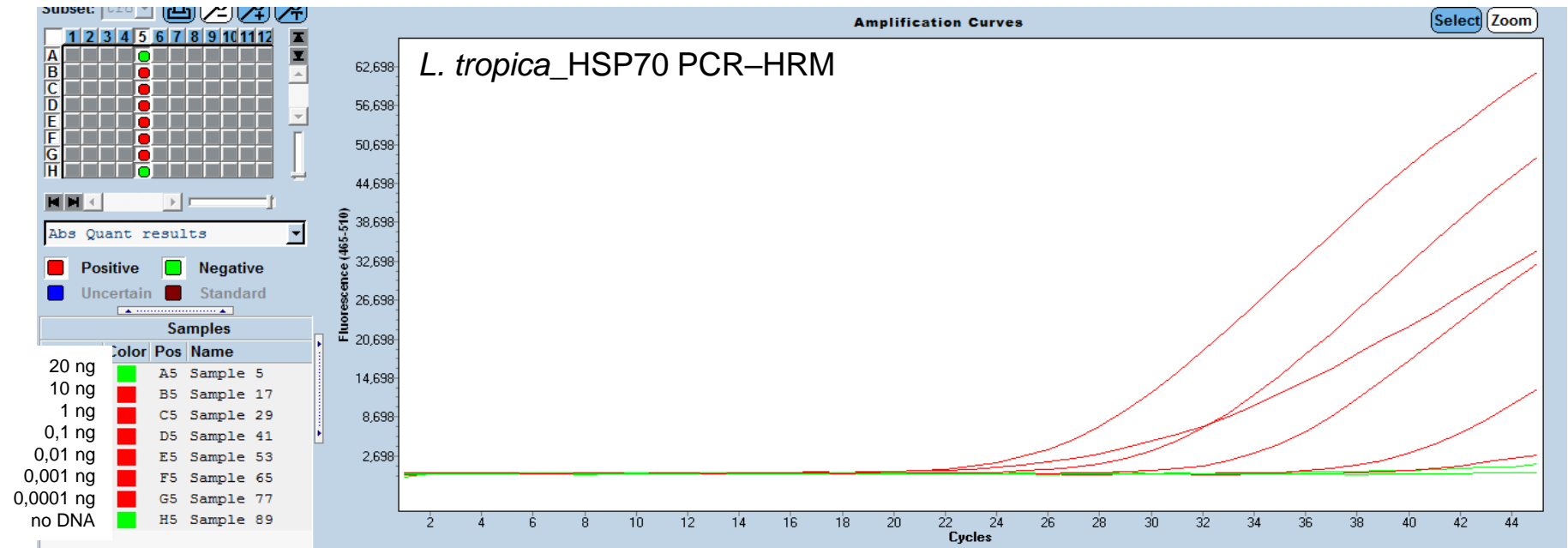

Supplement: Supplementary file 8 — Additional file 8: Figure S3. Sensitivity of PCR-HRM targeting 7SL RNA and HSP70 genes. Detection limit of PCR-HRM using 7SL RNA and HSP70 genes was determined using tenfold dilutions of DNA from each reference Leishmania isolate, representing L. infantum (MHOM/TN/94/LV50), L. major (MHOM/TN/2011/EMPA10), and L. tropica (MHOM/IQ/65/L75). a. 7SL PCR-HRM using L. infantum DNA b. HSP70 PCR-HRM using L. infantum DNA. c. 7SL PCR-HRM using L. major DNA d. HSP70 PCR-HRM using L. major DNA. e. 7SL PCR-HRM using L. tropica DNA f. HSP70 PCR-HRM using L. tropica DNA. The right sections of the figures show the tested samples, which are color-coded according to the amplification result, red when positive and green when negative. In all panels, sample codes correspond to the following DNA amounts: A: 20 ng; B: 10 ng; C: 1 ng; D: 0.1 ng; E: 0.01 ng; F: 0.001 ng; G: 0.0001 ng; H: negative control (no DNA). [file 13071_2021_5138_MOESM8_ESM.pdf]
